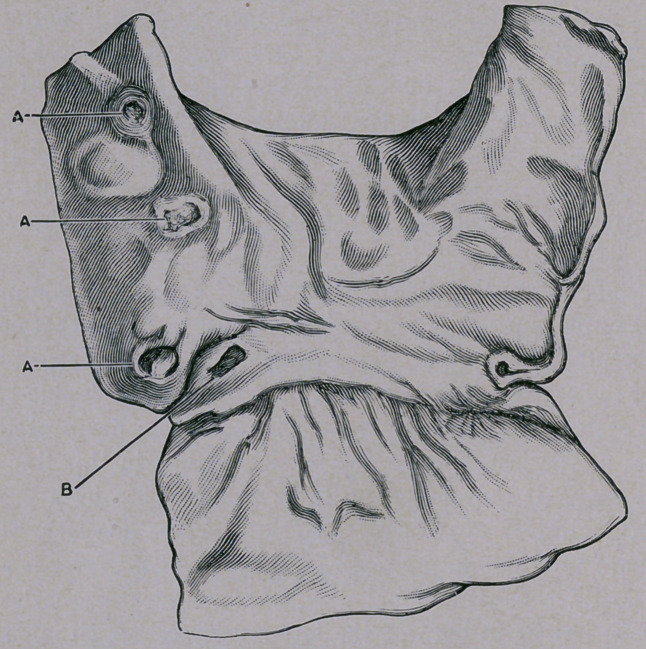# Typhoid Fever as a Systemic Disease of Manifold Manifestations

**Published:** 1899-08

**Authors:** 


### Typhoid Fever as a Systemic Disease of Manifold Manifestations.

It has become so common to regard typhoid fever as a local affection, the lesions of which are situated in the lower part of the small and the beginning of the large intestine, that the essentially systemic character of the disease has been more or less lost sight of. Of course, it is clearly understood that the absorption of toxines from the typhoid ulcers in the bowel gives rise to very prominent constitutional symptoms, while the occurrence of spots on the skin show a cutaneous attempt at one stage of the disease at least to eliminate certain toxic substances, biological or chemical in nature, from the circulation.

Most of the pharmaceutic schemes of treatment planned for typhoid fever, however, are limited to the use of drugs that act upon the intestinal tract. Intestinal antisepsis has been a favorite catchword of the ambitious therapeutist in many diseases beside typhoid, though each new attempt to create this condition has proved as ineffectual as the last, further therapeutic claims in this line gain a ready hearing if they but seem to be bolstered up by a pretended successful clinical experience. Of late years, however, we have come more and more to the realization that typhoid fever is as characteristically a constitutional disease as measles or scarlet fever. The main lesions in both of the latter diseases are situated in the upper air passages, but we by no means consider that the angina of scarletina or the severe coryza in measles constitutes the essence of either disease or furnishes the only indications for treatment. Prof. Chiari's work at Prague has shown that typhoid not infrequently limits itself to the bile passages and this notwithstanding all that we have recently learned about the bactericidal power of bile. Osler's work in this country, besides confirming Chiari's observations as to typhoid localization in the bile passages, has served to show that, exceptionally at least, the lesions of typhoid fever are limited to other localizations—the spleen for example. Certain French clinicians claim to have observed typhoid fever of the meninges or a fibrile disease in which the only possible cause discoverable was the presence of Eberth's or Jaffky's bacillus on these membranes.

Even where the lesions of typhoid fever are limited to the digestive tract we are gradually being brought to realize that they need not necessarily be localized within the immediate neighborhood of the cæcum, but under special conditions of poorly resistive vitality typhoid ulcers may occur in other parts of the gastro-intestinal tract. A striking illustration of this kind is afforded by one of the plates in Prof. Hare's new book on "The Medical Complications of Typhoid Fever."* The illustration which we present herewith reproduces a set of typhoid ulcers that had developed in the stomach of a young girl who succumbed during the third week of her attack. Four well-defined ulcers were noted in the pyloric region, one of which presented a loosely adherent slough. It appears that under certain circumstances, not well understood as yet, a diseased condition of the solitary glands of the gastric mucosa may give rise to a form of perforating ulcer of the stomach which closely resembles the idiopathic ulcer of typhoid fever as that lesion is usually observed in the ileo-colic region of the intestine. It is interesting to

*The Medical Complications, Accidents and Sequelæ of Typhoid or Enteric Fever, by H. A. Hare, M. D., with a special chapter on the Mental Distubances following Typhoid Fever, by F. X. Dercum, M. D. Lea Brothers & Co., Philadelphia and New York. 1899.

note that in this case there was no haematemesis to arouse suspicion of ulcers of the stomach.

The great diversity of typhoid complications has by no means received the general attention the importance of the subject deserves, and we venture to predict that by this more diligent study many dark places in both diagnosis and treatment will be made plain. In this direction Dr. Hare, with characteristic energy, has taken a long step.

## Figures and Tables

**Figure f1:**